# Unselective Transport of Phytopathogenic *Fusarium* Fungi from Litter and Soil by Ground-Dwelling Arthropods Links Semi-Natural and Agricultural Habitats

**DOI:** 10.3390/microorganisms10020335

**Published:** 2022-02-01

**Authors:** Nadja Heitmann, Michael Glemnitz, Klaus Birkhofer, Marina E. H. Müller

**Affiliations:** 1 Leibniz Centre for Agricultural Landscape Research (ZALF), Eberswalder Str. 84, 15374 Müncheberg, Germany; mglemnitz@zalf.de (M.G.); mmueller@zalf.de (M.E.H.M.); 2 Department of Ecology, Faculty of Environment and Natural Sciences, Brandenburg University of Technology, Cottbus-Senftenberg, Konrad-Wachsmann-Allee 6, 03046 Cottbus, Germany; klaus.birkhofer@b-tu.de

**Keywords:** insect-vector, ground-dwelling arthropods, *Fusarium*, phytopathogenic fungi, mycobiota, wheat, linkage, community

## Abstract

The dispersal of propagules, such as fungal spores or seeds by actively moving animals, connects and shapes communities. The dispersal of plant pathogens by arthropods might be a crucial mechanism in the spread of several crop diseases. Ground-dwelling arthropods are potential linkers between fungal communities in semi-natural and agricultural habitats by transporting propagules of *Fusarium* fungi. We compared the *Fusarium* communities on the body surface of ground-dwelling arthropods with litter in semi-natural and soil in agricultural habitats with a focus on the *Fusarium* community. We found three relatively distinct *Fusarium* communities with moderate overlap. We detected a higher richness of *Fusarium* species on the body surface of arthropods compared to litter and soil communities. The results suggest that the *Fusarium* community on the body surface of arthropods relates to the composition observed in litter and soil with limited filtering mechanisms between communities. Ground-dwelling arthropods are relevant agents for the distribution of *Fusarium* and therefore link fungal communities in adjacent habitats.

## 1. Introduction

The dispersal of propagules, such as spores or seeds by actively moving vertebrates or invertebrates, shapes ecosystems and communities in different ways, which is defined by the mobile link concept [1]. By moving actively within semi-natural habitats and through spillover into adjacent arable habitats, animals can introduce species or genes. Such passive dispersal links fungal communities but may also promote the spread of organisms that provide ecosystem disservices, such as phytopathogenic fungi. The dispersal of propagules from plant pathogens by arthropods might be a crucial mechanism for the spread of several crop diseases. It can enhance the spread of the pathogen and the disease development, shown for the kernel rot of maize (*Zea mays* L.) or Fusarium head blight (FHB) in wheat (*Triticum aestivum* L.), or the laurel wilt disease of avocados (*Persea americana* Mill.) [2,3,4].

One of the major pathogens in wheat production is fungi of the genus *Fusarium*. Nineteen *Fusarium* (*F*.) species are involved in the crop disease Fusarium Head Blight, *F. culmorum* (W.G. Smith) Saccardo, and *F. graminearum* Schwabe, which are among the most pathogenic species [5]. A reduced wheat grain size and quality as well as the production of mycotoxins are the results of this disease [6]. The composition of *Fusarium* communities and the severity of the disease in the crop are both affected by the fungal community in primary inoculum, which can arise from crop or weed plants, plant residuals, or soil [7,8]. The *Fusarium* community associated with weeds and their litter within crop fields and surrounding field margins is very diverse and consists of multiple *Fusarium* species such as *F. graminearum, F. verticillioides* (Saccardo) Nirenberg, *F. oxysporum* Schlechtendahl emend. Snyder & Hansen, *F. culmorum*, or *F. subglutinans* (Wollenweber & Reinking) Nelson, Toussoun & Marasas [8,9]. Weed species, especially grasses, can act as alternative hosts for *Fusarium* species and may harbor pathogenic fungal strains for cereal crops [10].

Ground-dwelling arthropods contact with fungal inoculum while moving through plant material or soil that is colonized by fungi [11,12]. Thereby, fungal propagules can attach to the body surface of arthropods (ectozoochory) [13]. Furthermore, many ground-dwelling arthropods such as carabid beetles or woodlice may hibernate in soil or seek shelter in small crevices or plant litter [14,15]. Fungal propagules can also adhere to the body surface while arthropods feed on colonized plant or soil material or on fungal mycelia or spores (fungivory), which is typical for many springtail species [16]. Numerous arthropod species of different taxa and trophic groups, such as herbivores, fungivores as well as predators are potential carriers of phytopathogenic fungal propagules, shown for ants, millipedes, and spiders [17]. Previous studies documented frequent external and internal transportation of multiple fungal taxa, including several species of the genera *Fusarium*, by ground-dwelling carabid beetles [18]. In general, fungal propagules can easily adhere to micro-structures such as bristles, hairs, and spines on the body surface of arthropods or the joints and mouthparts [19].

Ground-dwelling arthropods are potential linkers between semi-natural and agricultural habitats by transporting propagules of *Fusarium* fungi. Due to their prevalent movement type and distinct ecology, they are very likely to get in contact with primary inoculum and propagules of *Fusarium* spp. when moving on the ground or feeding on weeds and their residuals. Ground-dwelling arthropods are a very abundant and diverse group, and individuals of many species move frequently between semi-natural and agricultural habitats [20]. Their role in the dispersal of *Fusarium* across habitat borders is unknown.

This study compares the total fungal load and *Fusarium* communities on the body surface of ground-dwelling arthropods, the litter layer of semi-natural habitats, and the soil of agricultural habitats. The study was conducted around kettle holes in agricultural landscapes, as these habitats provide an abundance of host plants in which residuals (litter) may act as a source for phytopathogenic fungi such as *Fusarium*. In agricultural landscapes, kettle holes act as important conservation hot spots for many arthropod taxa such as carabid beetles or spiders [21,22].

**Hypothesis** **1.** *The total fungi and Fusarium load of the body surface of ground-dwelling arthropods is determined by the total fungal and Fusarium abundance in soil (for arable fields) or litter (for semi-natural habitats).*

**Hypothesis** **2.** *The species richness of Fusarium communities on the body surface of ground-dwelling arthropod is higher than in the soil or litter since arthropods move in both habitats.*

**Hypothesis** **3.** *The composition of Fusarium species in terms of relative abundances on the body surface of ground-dwelling arthropods is similar to that in soil (for arable fields) or litter (for semi-natural habitats).*

## 2. Materials and Methods

### 2.1. Study Site

The study site is located approx. 90 km north of Berlin in the Uckermark region in the Federal State of Brandenburg, Germany, in the Lowlands in North Germany, (GPS coordinates of the study area: between Fürstenwerder (53°23′19″ N 13°35′2″ E) and Prenzlau (53°19′2″ N 13°51′48″ E)). The agricultural landscape laboratory Quillow (AgroScapeLab) of the Leibniz Centre for Agricultural Landscape Research [23], in which this study was carried out, is located within this region. The subcontinental climate in this area shows a long-term mean annual temperature of 8.6 °C and average long-term annual precipitation of 564 mm (ZALF field station, Dedelow). The area represents typical landscapes in Central continental Europe that are formed by glaciations of the Pleistocene and post-glacial processes. Kettle holes, which are small water bodies surrounded by semi-natural vegetation margins, were created by Pleistocene processes in a high number [24]. 

### 2.2. Sampling Design

**Figure 1:**
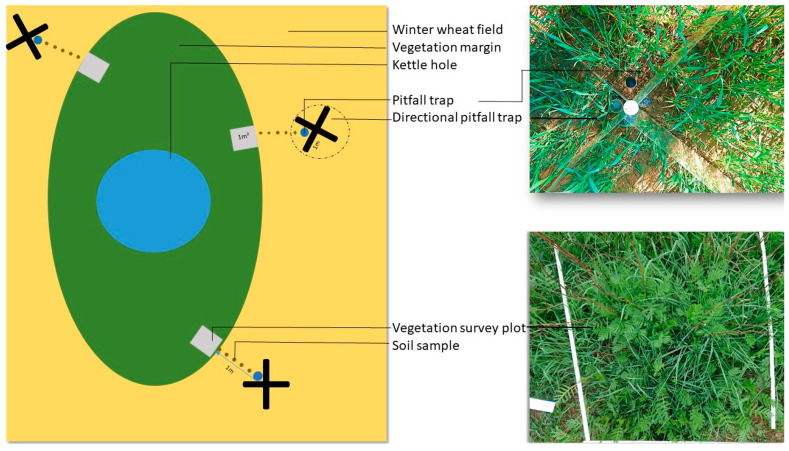
Sample design for the collection of arthropods, litter, and soil samples for the comparison of the associated fungal community. Seven kettle holes were sampled in total.

We analyzed the fungal community associated with ground-dwelling arthropods, soil, and litter at seven kettle holes surrounded by winter wheat fields. Kettle holes were selected according to their permanent presence of water during the year, size of their water body (min. 25 m^2^), their distances (at least 50 m) to field borders, roads, or other landscape elements, and the vegetation of their margins (dominated by grasses). Each kettle hole was sampled at three replicated sampling points (A, B, and C) that were at least 8 m apart from each other and 1 m into the crop field (Figure 1). Sampling points were placed into grassy margins, with at least 10 m distance to any trees or shrubs.

We collected ground-dwelling arthropods at each sampling point with a system of directional pitfall traps. Two transparent acrylic glass plates (2 m × 0.25 m) were inserted upright ca. 2 cm into the ground in a cross-design. The glass plates acted as a barrier to direct arthropods into pitfall traps (glass jars, upper diameter: 6.5 cm) that were inserted into the ground at each of the four corners of the directional pitfall trap (Figure 1). For this study, only arthropods that were collected with the pitfall trap facing the edge of the kettle holes were analyzed in order to study beetles that moved from the vegetation of the kettle hole into the crop field. The traps were operated between 25 and 26 May 2020 for 24 h without any collecting or preservation fluid to avoid the displacement or damage of fungal propagules. All arthropods were sorted individually in 2 mL Eppendorf tubes after collecting and were stored at 5 °C in darkness overnight after sampling. 

Samples were then analyzed by the microbial culture-dependent method within 24 h, described under paragraph “2.3. Fungal load on the body surface of arthropods and fungal abundances in soil and litter”. After the microbial analyses, the arthropods were stored in 70% ethanol and taxonomically determined following the script of Stresemann [25]. The arthropod taxa and their number of individuals used in the analyses are listed in Table S1.

The vegetation of the margins surrounding the kettle holes was sampled by establishing 1 m × 1 m plots at each sampling point beginning at the edge of the crop field (Figure 1). A vegetation survey prior to the arthropod sampling was conducted on the same day. The total surface cover by vegetation (%) and mean height (cm), the relative cover of grasses (%), as well as the share of dead plant biomass on total biomass (%), were visually estimated following the Braun-Blanquet approach (Table S2). Within the 1 m × 1 m plots, litter samples consisting of circa 100 g of dead and living plant parts up to a height of 10 cm were collected on 25 May 2020 based on the previously conducted vegetation survey. Soil samples (circa 100 g) were taken at each sampling point on 24 May 2020. The soil of the upper 3 cm was sampled across lines between the vegetation margin of the kettle hole and the position of the pitfall traps in the winter wheat fields. We assume that fungi in deeper soil layers do not relate to the fungal community on arthropods moving over the soil and would therefore not explain patterns of fungal abundance and *Fusarium* communities on the arthropods. Five soil samples along every line were mixed into one bulk soil sample at each sampling point. The dry mass of the litter and soil samples was determined by drying a subsample of 5 g of each sample for 24 h at 105 °C for soil samples and 48 h at 60 °C for litter samples. The fresh and dry weights of the samples were compared to calculate the dry mass. 

### 2.3. Fungal Load on the Body Surface of Arthropods and Fungal Abundances in Soil and Litter

We quantified viable propagules of fungi in general (total fungi) and fungi of the genus *Fusarium* attached to the body surface of arthropods (exogenous) as well as in the soil and the litter samples. Arthropods were stored for 20 min at −20 °C. The 1.1 mL quarter-strength Ringer‘s solution with 0.1% Tween 80 was added to one arthropod per one Eppendorf tube. Arthropod samples were then placed into an over-head shaker for 2 min at 30 rpm at room temperature to remove fungal propagules from the body surface via a washing process, according to Heitmann et al. 2021 [18]. A subsample of 5–7 g of plant material was selected from each litter sample. The plant material was added to 180 mL sterile aqua dest and treated for 2 min in the Stomacher 400 Circulator (Seward Ltd., Thetford, United Kingdom), according to Müller et al. 2018 [26]. Then, 10 g of the fresh soil samples were immersed in 90 mL sterile pyrophosphate solution (0.1% sodium pyrophosphate + 0.8% sodium chloride). Five grams of sterilized natural stones (diameter ca. 0.5 cm) were added to the soil samples to break up soil aggregates. Samples were placed in an overhead shaker for 30 min at 30 rpm.

Suspensions of the arthropods, litter, and soil samples were spread onto Czapek Dox Iprodion Dichloran agar (CZID) [27], for litter and soil in a decimal dilution series using quarter-strength Ringer’s solution with three replicate Petri dishes (diameter 9 cm) each. For the arthropod samples, 0.2 mL and 0.3 mL of the undiluted suspension were spread on two Petri dishes, two replicates each. All Petri dishes were incubated at 25 °C for 3 days without light followed by 2–4 days under mixed black UV light (emission ca. 310–360 nm) and with a photoperiod of 12:12 h of artificial daylight at room temperature. All germinated fungal propagules were counted as fungal colonies (colony forming units, CFU). *Fusarium* fungi were identified on the genus level based on colony morphology. The total fungal abundance and the *Fusarium* spp. abundance were expressed per arthropod individual (TOTAL indiv^−1^, FUS indiv^−1^) and per 1 g dried litter and soil (TOTAL g^−1^ DM, FUS g^−1^ DM). 

*Fusarium* colonies were transferred onto potato dextrose agar (PDA, Carl Roth GmbH Karlsruhe, Germany) as well as Synthetic Nutrient-Poor Agar (SNA; [28]) for taxonomic identification to the species level and incubated as described before. Identification was carried out according to the guidelines of Leslie and Summerell [29] and Yli-Mattila et al. [30]. *Fusarium* fungi were isolated from all Petri dishes of the arthropod samples, from the 10^−4^ dilution of the litter samples and the 10^−3^ dilution of the soil samples. The number of germinated CFU on Petri dishes of these dilution levels was sufficiently high to detect rare *Fusarium* species and thus provide an overview of the *Fusarium* communities. At the same time, the number of CFU of *Fusarium* and other fungi was low enough to obtain uncontaminated single spore germination cultures of *Fusarium* fungi. The mean number of CFU per Petri dish was 196 for the 10^−4^ dilution of the litter samples and 79 for the 10^−3^ dilution of the soil samples. The resulting data were the basis for the comparison of the *Fusarium* species composition in the community analysis.

### 2.4. Statistical Analysis 

The *Fusarium* species richness and dominance structure, as well as the total fungal and *Fusarium* abundance in soil and litter (number of colony forming units (CFU) per 1 g dry mass) and the load on the arthropods (CFU per individual), respectively, were analyzed in adapted statistical approaches. In a three factorial design, the factors “substrate type” (arthropod, litter, soil), “kettle hole” (7 kettle holes), and “sample point” (1–15) were defined. The factors “kettle hole” and “sampling point” were important due to the spatial dependency of arthropod, litter, and soil samples in the design. Note that 6 sampling points at kettle holes did not yield arthropod samples. 

For all statistical analyses, the data of the arthropod samples were averaged across all individuals per sampling point. Abundance data of total fungi and *Fusarium* fungi were transformed (ln(x + 1)), and the abundance of the *Fusarium* species were standardized to dominances (0–100%) for each sampling point. All statistical analysis were performed in R 4.1.1 [31]. Permutational analyses of variances (PerMANOVA) with the function “adonis2” in the package “vegan” were used to analyze differences in the taxonomic composition of *Fusarium* communities, followed by pair-wise analyses with the function “pairwise.adonis2” of the package “pairwiseAdonis” comparing pairs of factor levels [32]. All factors were included in the PerMANOVA as fixed factors. All resemblance matrices were based on Gower similarities between communities and were analyzed performing 9999 permutations with the permutation method ‘‘Permutation of residuals under a reduced model’’ [32]. To compare the three *Fusarium* communities visually, the effects of the factor “substrate type” on the taxonomic composition in terms of the dominance of species were visualized with nonmetric multidimensional scaling (nMDS) with the “ggvegan” package. To analyze the contribution of each fungal species to dissimilarities between the fungal communities of the different substrate types, a SIMPER analysis based on the Bray-Curtis dissimilarity index was performed with the function “simper” in the package “vegan”.

To unravel potential relationships between the total fungal and *Fusarium* load on the body surface of arthropods and the total fungal and *Fusarium* abundance in soil and litter, a Spearman correlation matrix was calculated with the basic function “cor” and the function “corrplot” of the “corrplot” package. To test for a potential relationship between the taxonomic composition of the arthropod samples on the species composition of *Fusarium*, a Mantel test was conducted with the function “mantel” in the package “vegan”.

## 3. Results

We collected 74 arthropods from six different orders on 15 out of 21 sampling points; Coleoptera was the most abundant taxa (Table S1). The body surface of 71 arthropod individuals had fungal propagules (colony forming units, CFU); the maximum was 8125 CFU per individual. Propagules of *Fusarium* spp. were detected on 41 of the 74 arthropods, up to 64 CFU per individual. All litter and soil samples contained propagules of total fungi and of *Fusarium* spp. Total fungal abundance was on average more than 36 times lower in the soil than in the litter (Table 1). The untransformed descriptive data on the 74 arthropod samples are presented in Table S3. 

### 3.1. Total Fungal and Fusarium Load on the Body Surface of Arthropods and Fungal Abundances in SOIL and Litter

**Figure 2:**
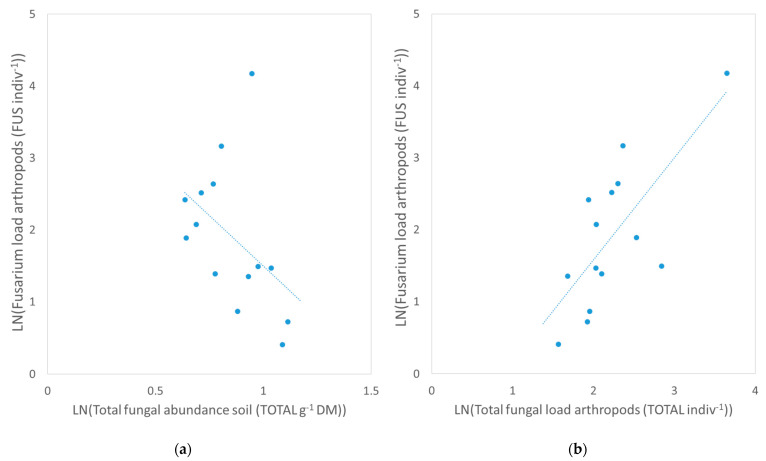
Relationship of the *Fusarium* load (FUS indiv^−1^) on the body surface of ground-dwelling arthropods to (**a**) the total fungal abundance (TOTAL g^−1^ DM) in the soil, (**b**) the total fungal load (TOTAL indiv^−1^) on the body surface of ground-dwelling arthropods detected as number of colony forming units (CFU) with culture-dependent method. All data are transformed LN(x + 1).

**Figure 3:**
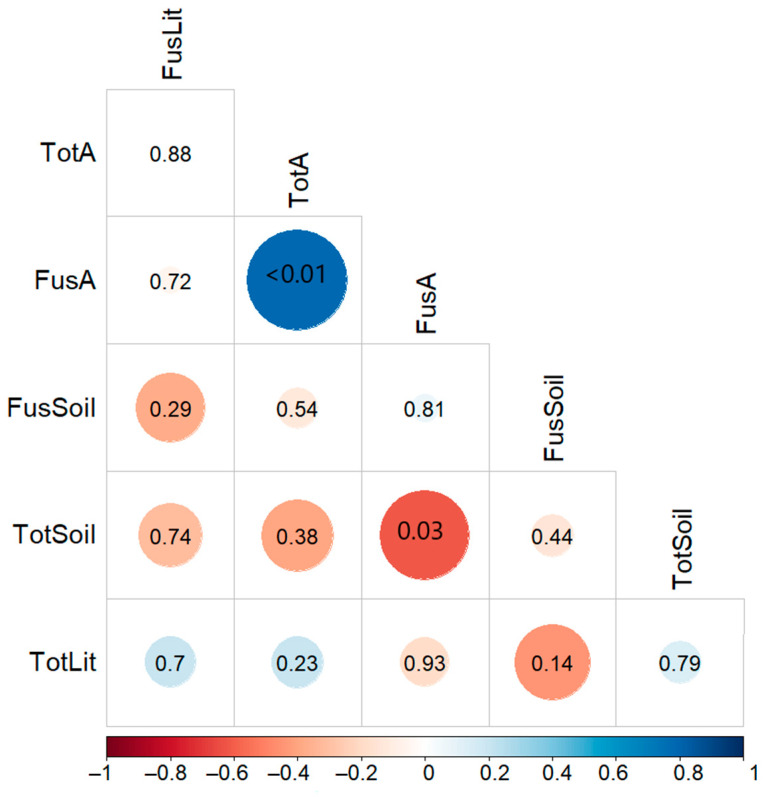
Correlation matrix with the Spearman correlation coefficients between the total fungal (Tot) and *Fusarium* (Fus) load on the body surface of arthropods (A), and abundance of *Fusarium* and total fungi in soil (Soil) and litter (Lit) samples, respectively. Darker colors and larger circles indicate stronger correlations. *p*-values are given for each correlation.

The *Fusarium* load on the body surface of arthropods correlated significantly and negatively with the total fungal abundance in the soil (r_sp_ = −0.61, *p* = 0.03, Figure 2a) and significantly and positively with the total fungal load on the arthropods (r_sp_ = 0.78, *p* < 0.001; Figure 2b). Arthropods with a high total fungal load also showed a high *Fusarium* load. No other significant correlations were detected between total fungal and *Fusarium* abundance or between fungal loads of different substrates (Figure 3). 

### 3.2. Fusarium Species Richness and Composition in Different Substrate Types

**Figure 4:**
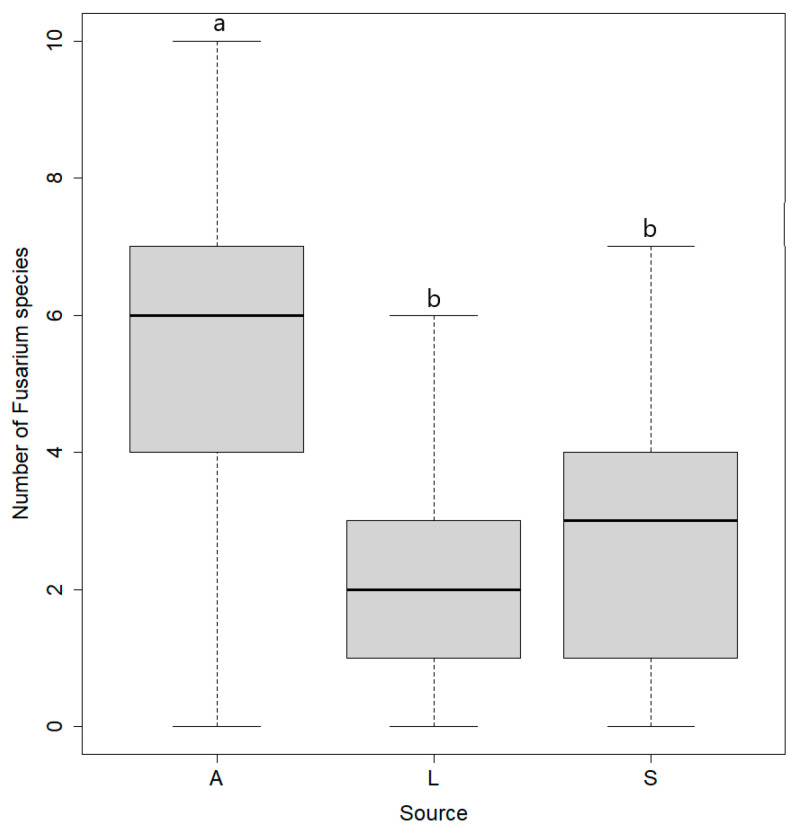
Number of identified *Fusarium* species on the body surface of arthropods (A), in samples of litter (L) and soil (S). The midline of all boxplots represents the median, with the upper and lower limits of the box being the third and first quartile, respectively. Whiskers will extend up to 1.5 times the interquartile range from the top/bottom of the box. Different letters above the boxplots indicate significant differences.

In total, 17 *Fusarium* species were detected from the body surface of arthropods and from litter and soil samples (Table S4). The substrate type significantly affected the *Fusarium* species richness (Table 2). The body surface of arthropods on average had significantly more *Fusarium* species per sample than observed in litter and soil samples. The species richness of litter and soil samples did not differ significantly (Figure 4). The *Fusarium* community on the body surface of arthropods consisted of 15 species in total. In the litter of the kettle hole vegetation, 12 species were observed and in the soil of wheat fields, 13 species were observed in total. 

**Figure 5:**
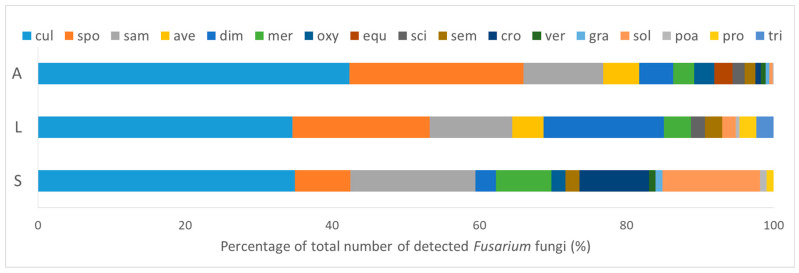
Dominance structure as percentage of each *Fusarium* (*F.*) species of the total number of detected *Fusarium* colony forming units (CFU), isolated from the body surface of ground-dwelling arthropods (A, CFU indiv^−1^), litter (L), and soil (S) (CFU g^−1^ DM). Species code: *F. culmorum* (cul), *F. sporotrichioides* (spo), *F. sambucinum* (sam), *F. avenaceum* (ave), *F. dimerum* (dim), *F. merismoides* (mer), *F. oxysporum* (oxy), *F. equiseti* (equ), *F. scirpi* (sci), *F. semitectum* (sem), *F. crookwellense* (cro), *F. verticillioides* (ver), *F. graminearum* (gra), *F. solani* (sol), *F. poae* (poa)*, F. proliferatum* (pro), *F. tricinctum* (tri).

*Fusarium culmorum* was the most common species in all three substrates and accounted for 42% of all detected *Fusarium* spp. on the arthropods (Figure 5). Table S4 shows the total sums of each identified *Fusarium* species detected on all 74 collected arthropods as well as in the litter samples at 10^−4^ and soil samples at 10^−3^ dilution levels. The mean abundances of each *Fusarium* species per sampling point are shown for the arthropods, litter, and soil samples in Tables S5–S7. *F. equiseti* was only found on the body surface of arthropods with a low percentage of 2.5% of all identified species. However, *F. equiseti* was present in five out 15 arthropod samples. *F. proliferatum* and *F. tricinctum* were not identified from the body surface of arthropods and were rarely observed in the litter and soil samples (Figure 5). *F. dimerum* was frequently isolated from litter samples, and accounted for 16.0% of all detected *Fusarium* spp. in the litter, but were rarely found in soil samples. However, *F. solani* and *F. crookwellense* showed the opposite pattern being often isolated from soil and accounted for 13.2% and 9.4%, respectively, of all detected *Fusarium* spp. in the soil (Figure 5). 

**Figure 6:**
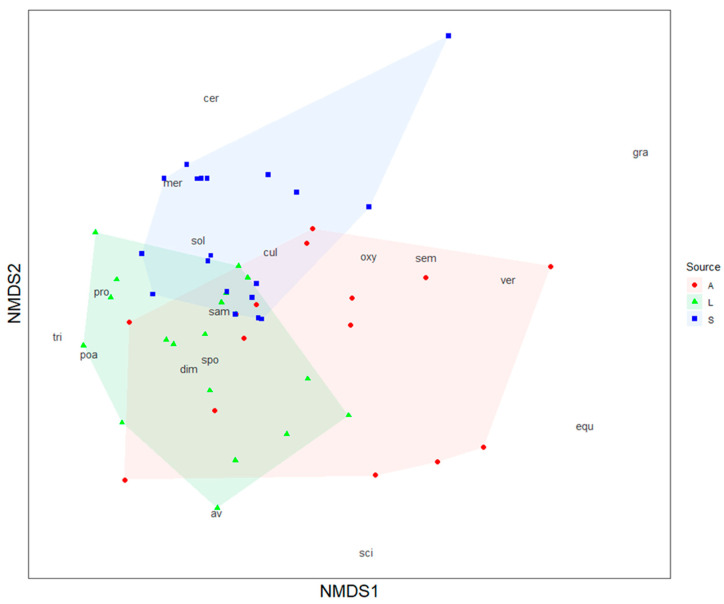
Non-metric multi-dimensional scaling (NMDS) ordination based on the Gower distance matrix of the *Fusarium* (*F*.) species dominance structure, isolated from the body surface of ground-dwelling arthropods (A), from litter samples of kettle hole margins (L), or soil from the adjacent winter wheat field (S). Resemblance in the dominance structure between the three substrate is shown in symbols. The symbols represent samples; samples that are very distinct from each other are further away in the plot. The abbreviations indicate *Fusarium* species. Species code: *F. avenaceum* (ave), *F. crookwellense* (cer), *F. culmorum* (cul), *F. dimerum* (dim), *F. equiseti* (equ), *F. graminearum* (gra), *F. merismoides* (mer), *F. oxysporum* (oxy), *F. poae* (poa), *F. proliferatum* (pro), *F. sambucinum* (sam), *F. scirpi* (sci), *F. semitectum* (sem), *F. solani* (sol), *F. sporotrichioides* (spo), *F. tricinctum* (tri), *F. verticillioides* (ver).

In general, the *Fusarium* species composition of different substrate types differed significantly in terms of relative abundances (Table 2). All three substrates showed a distinct composition of *Fusarium* species based on their relative abundances, but the ordination also indicates moderate overlap between arthropod and litter communities (Figure 6). The most influential species for discriminating the *Fusarium* communities between pairs of substrate types were *F. sporotrichioides*, *F. culmorum*, and *F. sambucinum* (Table 3). The composition of the analyzed arthropod taxa at each sampling point was not significantly related to the species composition of *Fusarium* communities on the body of arthropods at each sample point (Mantel: R = −0.04, *p* = 0.55).

## 4. Discussion

Viable propagules of different fungal genera were detected on the body surface of most of the investigated arthropods (96%). Viable *Fusarium* propagules were also detected on the majority of arthropods (55%). These results suggest that ground-dwelling arthropods frequently interact with fungi of different taxa and that fungal propagules stay viable on the body surface of the arthropods. In an experimental study, propagules of *F. acuminatum* Ellis & Everhart stayed viable on an insect vector for 96 h after exposure [33]. Arthropods have a high potential to act as vectors for propagules of *Fusarium* spp. and other fungal genera. Considerably lower frequencies of phytopathogenic fungi were detected on arthropods potentially vectoring for the Fusarium wilt of banana, caused by *F. oxysporum*, and the vine trunk disease [17,34].

The positive correlation between the *Fusarium* load and the total fungal load on the arthropods was strong (r_sp_ = 0.78), which coincides with a previous study on ground-dwelling carabid beetles [18]. Fungi of the genera *Fusarium*, *Penicillium*, *Epicoccum*, *Cladosporium*, *Aspergillus*, and yeasts such as *Candida* are commonly found on the surface of arthropods [35,36,37]. The strong correlation between total fungi and *Fusarium* fungi suggests that no selective mechanism exists that either reduces or promotes the attachment of *Fusarium* propagules to the body surface of arthropods in comparison to other fungal propagules. In general, volatile organic compounds (VOCs) can be recognized by arthropods and play an important role in their orientation and predation behavior [38,39]. The VOCs emitted by plants infected by *Fusarium* fungi can be attractive to some arthropods that vector these pathogens and change their behavior accordingly [40,41]. However, these microbially produced volatiles can also be repellant for other arthropods, and the mycotoxins produced by the *Fusarium* fungi can reduce the fitness of arthropods [39,42].

The *Fusarium* load on the arthropods only correlated significantly with the total fungal abundance in the soil of the crop field (r_sp_ = −0.61), supporting our first hypothesis, which expected an effect of the litter and soil fungal abundance on the fungal load of the arthropods partly. No relationships were detected between the total fungal load of the arthropods and the soil, or between the litter and soil. This suggests that other factors affect the total fungal and *Fusarium* distribution, which were not investigated in this study, such as soil humidity, air temperature, or management practices. Previous studies show that a humid and cold micro-climate increases the abundance of *Fusarium* spp. but decreases the abundance of *Alternaria* spp., another common phytopathogenic fungi, and that the application of residual manure and crop rotation affects the microbial composition in maize fields [43,44]. Further studies should aim for a metabarcoding analysis of the mycobiome in the soil, litter, and on the arthropods to identify the entire fungal community at a species level. Including abiotic and biotic environmental factors of the soil and adjacent semi-natural habitats such as soil moisture in further studies would help to detect relationships between the fungal loads of potential vectors and the fungal abundance in their habitats. Further studies should include the soil in the semi-natural habitats, as well as wheat plants and the crop residuals in the arable fields since they are an important inoculum source for *Fusarium* spp. [8]. A contamination of the external fungal load of the arthropods with fungal propagules from the feces or other body secretes cannot be excluded and should be addressed in further studies.

The body surface of arthropods had more *Fusarium* species (in total and on average) than in litter and soil samples, which confirms our second hypothesis. The species composition of *Fusarium* communities on arthropods consisted of species found in litter samples of the kettle hole margins as well as in the soil of the arable field. These results indicate that arthropods may exchange fungal propagules between the semi-natural habitats and the arable fields. *F. equiseti* was unique for arthropods and was detected frequently in low concentrations. This indicates that arthropods contact with fungal propagules of *F. equiseti* at crop residuals in the crop field or in areas closer to the kettle hole, which were not sampled in this study. In other studies, *F. equiseti* was regularly detected on ground beetles, soil, and senescent or damaged plant tissue [18,29]. *F. proliferatum* and *F. tricinctum* were present in the litter or soil samples in low frequencies and concentrations. Therefore, it is very likely that they were simply not detected on the body surface of arthropods due to their local rarity. In general, the most abundant *Fusarium* species *F. culmorum*, *F. dimerum*, *F. sambucinum*, *F. sporotrichioides*, and *F. solani* were also commonly found on the body surface of the investigated arthropods. The results imply that the transport of *Fusarium* propagules by ground-dwelling arthropods is not selective and is not limited to certain *Fusarium* species in terms of adhesion as well as survival on the body surface of the arthropods. If species are missing on the body surface of arthropods, they are rare in soil and litter, and if species are only present on the body surface, they were probably introduced from other habitats.

Fusarium head blight (FHB) is one of the most important diseases in wheat production and is caused by a complex of 19 *Fusarium* species [5,6]. Within this complex, species differ in their ecology, virulence, and mycotoxin production and therefore in their importance for the development of FHB and its economic consequences [6]. *F. culmorum* is one of the main agents of FHB and accounted for a major proportion of the *Fusarium* spp. identified here. Therefore, these results might be relevant to the management of this plant disease. *F. culmorum* has already been identified on ground beetles, wheat stem sawflys (*Cephus cinctus* Norton), and is commonly found in soil and on plant debris [18,29,45]. All *Fusarium* species detected in this study were morphologically determined and were based on viable exogenous propagules. Therefore, a genetic approach that examines the entire fungal load of arthropods (endogenous and exogenous) and distinguishes *Fusarium* species by genetic traits should be addressed in further studies.

*Fusarium* fungi produce different types of propagules, which can be detected with culture-dependent methods. Some *Fusarium* species can produce smaller, often unicellular microconidia in addition to sickle-shaped, multicellular macroconidia [29]. Chlamydospores, which are thick-walled large resting spores, and the sexually produced ascospores are also typical for some *Fusarium* species [29]. Propagules of *Fusarium* fungi are commonly distributed by wind, rain splash, or by arthropods [33,46,47]. However, a previous study investigating the dispersal of spores of different *Fusarium* species by wind suggests a combination of the different dispersal mechanisms [48]. The different spore types of the *Fusarium* fungi did not affect the probability to adhere to the body surface of the arthropods in this study. Analyses at the species level of the entire fungal community in the soil, litter, and on the body surface of the arthropods could provide information on whether this is also the case for other fungal genera.

The results contradict the third hypothesis; the *Fusarium* species composition in terms of relative abundances on the body surface of the arthropods differed from that in soil and litter. *F. culmorum* occupied a considerably larger proportion in the *Fusarium* community on the arthropods than in soil and litter. Most *Fusarium* species were present in all three substrates, as described before. However, they differed in their relative abundance, so that the arthropods, litter, and soil samples displayed distinct *Fusarium* communities. Semi-natural habitats as well as crop fields are crucial habitats for ground-dwelling arthropods, and they can move between both habitat types, e.g., via spillover effects [14,20]. Arthropods interact with fungi in both the soil of the fields and the litter of the kettle holes, both of which had distinct fungal communities in this study. Therefore, the distinct *Fusarium* community on the arthropods probably results from an interaction with *Fusarium* communities in both habitats. The NMDS revealed more similarities between the arthropod and the litter *Fusarium* community than with the soil. This could be explained by the high importance of semi-natural habitats for many animal species such as ground beetles, spiders, and bees [22,49]. The experimental design, placing the traps 1 m away from the margin of the kettle hole, probably also influenced the results. However, it also showed a clearer picture of the fungal load of the arthropods leaving the kettle hole and possibly spreading fungal propagules into the crop fields. Nevertheless, the arthropods’ *Fusarium* community also showed similarities with the soil of the crop field, but the overlap was much smaller. Sampling arthropods within crop fields at different distances from semi-natural habitats could improve the understanding of the relationship between the fungal community on the arthropods and the environmental fungal communities.

## 5. Conclusions

Ground-dwelling arthropods revealed a remarkable potential to spread phytopathogenic and other fungi into arable fields. They interacted with the *Fusarium* community in the litter of semi-natural habitats as well as in the soil of adjacent crop fields, displaying a distinct and very species-rich *Fusarium* community. Exogenous dispersal by arthropods did not favor or hinder spores of specific *Fusarium* species. These results might be important for understanding fungal community dynamics and spatiotemporal disease patterns in crop fields. In addition to other dispersal mechanisms such as wind, arthropods could lead to a link between micro-communities and habitats. Further studies should analyze a broader range of fungal species by using a metabarcoding approach and by sampling arthropods within crop fields at different distances from semi-natural habitats. This could provide more insights about how the fungal community on the arthropods relates to environmental fungal communities.

## Figures and Tables

**Table 1:** Descriptive data of quantified total fungal (Tot) and *Fusarium* (Fus) colony forming units from the body surface of collected arthropods (Arthrop (Indiv^−1^), and from litter and soil samples (g^−1^ DM).

	Arthrop (Indiv^−1^)		Litter (g^−1^ DM)		Soil (g^−1^ DM)	
	Tot	Fus	Tot	Fus	Tot	Fus
Min.	23.80	0.00	64.01	0.39	4.33	0.05
1st Qu.	85.27	2.12	124.51	2.38	5.99	0.21
Median	107.85	3.44	257.87	3.83	7.48	0.35
Mean	452.47	9.96	300.09	7.12	8.23	0.36
3rd Qu.	215.67	10.78	406.18	7.80	9.46	0.45
Max.	4447.00	64.00	755.80	47.67	14.92	0.93

**Table 2:** Effects of the different kettle holes, sampling points and substrate types arthropods (A), litter (L), soil (S) on the species composition of *Fusarium* communities based on relative abundances (Dominance) and the number of identified *Fusarium* species (Richness) in (A) the global model and (B) pairwise post-hoc tests. Significant *p*-values from PerMANOVA are shown in bold.

		Dominance		Richness	
(A) Global Model	df	F	*p*	F	*p*
Kettle hole	6	1.24	0.143	1.1	0.371
Sampling point	14	1.08	0.305	1.19	0.328
Substrate type	2	4.9	**<0.001**	12.29	**<0.001**
**(B) Pairwise Comparison**					
A × L	1	3.24	**0.002**	24.41	**0.001**
A × S	1	4.46	**0.001**	16.84	**0.002**
S × L	1	6.05	**0.001**	0.67	0.492

**Table 3:** Cumulative percentage contribution (Cum. Con. Up to a threshold of 50%) to the difference between communities by the *Fusarium* species that differentiate most between pairs of substrate types according to similarity percentage analysis.

Arthropods vs. Litter		Arthropods vs. Soil		Litter vs. Soil	
Species	Cum. Con	Species	Cum. Con	Species	Cum. Con
*F. sporotrichioides*	0.23	*F. culmorum*	0.24	*F. culmorum*	0.22
*F. culmorum*	0.42	*F. sambucinum*	0.39	*F. sporotrichioides*	0.43
*F. dimerum*	0.57	*F. dimerum*	0.52	*F. sambucinum*	0.56

## Data Availability

The data presented in this study are available on request from the corresponding author.

## Supplementary Materials

The following are available online at www.mdpi.com/2076-2607/10/2/335/s1, Table S1: Number of collected individuals (N indiv) of different arthropod orders, Table S2: Vegetation survey of seven kettle hole margins of 21 plots [50], Table S3: Untransformed descriptive data of the quantified total fungal and *Fusarium* load (CFU Indiv^−1^) of 74 collected arthropods, Table S4: Number of colony forming units (CFU) of *Fusarium* species detected on the body surface of 74 collected arthropods, Table S5: Dominance structure as number of colony forming units of *Fusarium* species detected on the body surface of collected arthropods, Table S6: Dominance structure as number of colony forming units of each *Fusarium* species detected in 1.0 mg dry mass of litter samples, Table S7: Dominance structure as number of colony forming units of *Fusarium* species detected in 1.0 mg dry mass of soil samples.
