# Supplementary material for: Unselective Transport of Phytopathogenic Fusarium Fungi from Litter and Soil by Ground-Dwelling Arthropods Links Semi-Natural and Agricultural Habitats
Source: Microorganisms. 2022 Feb 1;10(2):335. doi: 10.3390/microorganisms10020335 (PMC8879785; doi:10.3390/microorganisms10020335)
Supplement: Supplementary file 1 [file microorganisms-10-00335-s001.zip › microorganisms-1554495-supplementary.pdf]

Supplementary Materials

Table S1. Number of collected individuals (N Indiv) of different arthropod orders and suborders per sampling point (A, B, C) at seven kettle holes. Sampling points with zero collected arthropods are not shown (1192A and B, 163A and B, 172B and C).

Sampling point	Order	Suborder	Common name	N Indiv
163C	Isopoda	Oniscidea	Woodlice	2
166A	Coleoptera	multiple	Beetles	5
	Isopoda	Oniscidea	Woodlice	1
166B	Coleoptera	multiple	Beetles	4
	Hemiptera	Heteroptera	Bugs	1
166C	Coleoptera	multiple	Beetles	4
172A	Isopoda	Oniscidea	Woodlice	1
	Julida	Julidae	Millipedes	2
807A	Coleoptera	multiple	Beetles	5
	Julida	Julidae	Millipedes	2
807B	Coleoptera	multiple	Beetles	5
807C	Coleoptera	multiple	Beetles	4
	Julida	Julidae	Millipedes	2
808A	Coleoptera	multiple	Beetles	4
	Dermaptera	Neodermaptera	Earwigs	1
	Entomobryomorpha	unknown	Springtails	2
	Isopoda	Oniscidea	Woodlice	3
808B	Coleoptera	multiple	Beetles	7
	Julida	Julidae	Millipedes	1
808C	Coleoptera	multiple	Beetles	6
1172A	Coleoptera	multiple	Beetles	1
	Isopoda	Oniscidea	Woodlice	2
1172B	Coleoptera	multiple	Beetles	3
1172C	Coleoptera	multiple	Beetles	5
1192C	Coleoptera	multiple	Beetles	1

Table S2. Vegetation survey of seven kettle hole margins of 21 plots (each 1 m x 1 m). The mean vegetation height in cm (Mean veg. height in cm), total vegetation cover in % (Total veg. cover %), the share of dead plant biomass on total biomass in % (Dead plant %), and the relative cover in % of gramineous weeds (Cover grasses %), were visually estimated following the Braun-Blanquet approach [1].

Kettle hole ID Sampling point	Mean veg. height (cm)	Total veg. cover (%)	Dead plant (%)	Cover grasses (%)
163A	100	100	20	50
163B	50	80	10	20
163C	90	80	30	110
166A	120	70	10	20
166B	50	100	10	30
166C	50	50	10	30
172A	50	40	50	90
172B	30	100	60	40
172C	50	90	10	60
807A	40	80	10	80
807B	40	90	20	80
807C	40	80	20	20
808A	40	90	70	40
808B	30	70	70	50
808C	40	80	70	70
1172A	20	90	20	20
1172B	50	40	20	20
1172C	30	60	50	50
1192A	20	90	20	20
1192B	50	100	70	40
1192C	50	90	60	90

Table S3. Untransformed (explained under 2.4 Statistical analysis) descriptive data of the quantified total fungal (Total fungi) and *Fusarium* load (*Fusarium*) in CFU Indiv^{-1} of 74 collected arthropods.

	Total fungi	*Fusarium*
Min.	0.00	0.00
1st Qu.	17.25	0.00
Median	56.13	1.00
Mean	313.84	6.00
3rd Qu.	149.50	6.00
Max.	8125.00	64.00
Total Occurrence Frequency in %*	95.95	55.40

* Frequency in percentage of the arthropods on which body surface fungal or *Fusarium* propagules were detected.

Table S4. Number of colony forming units (CFU) of *Fusarium* species detected on the body surface of 74 collected arthropods per individual ($Indiv^{-1}$), and in dry mass (DM) of 21 litter (mg^{-1} DM) and 21 soil samples (mg^{-1} DM), summed up for all samples per substrate type. Abbreviations are used in Figure 5 and 6 and Tables S5-S7.

***Fusarium* species**	**Ab-bre-via-tion**	**Arthropods CFU Indiv^{-1}**	**Litter CFU mg^{-1} DM**	**Soil CFU mg^{-1} DM**
Fusarium avenaceum (Fries) Saccardo	Ave	19.00	90.00	0.00
Fusarium crookwellense Burgess, Nelson & Toussoun	Cer	2.00	0.00	10.00
Fusarium culmorum (W.G. Smith) Saccardo	Cul	146.25	740.00	37.00
Fusarium dimerum Penzig	Dim	26.00	350.00	3.00
Fusarium equiseti (Corda) Saccardo	Equ	13.00	0.00	0.00
Fusarium graminearum Schwabe	Gra	4.00	0.00	1.00
Fusarium merismoides Corda	Mer	5.00	80.00	8.00
Fusarium oxysporum Schlechtendahl emend. Snyder & Hansen	Oxy	8.50	0.00	2.00
Fusarium poae (Peck) Wollenweber	Poa	1.00	10.00	1.00
Fusarium proliferatum (Matsushima) Nirenberg	Pro	0.00	50.00	1.00
Fusarium sambucinum Fückel	Sam	46.75	240.00	18.00
Fusarium scirpi Lambotte & Fautrey	Sci	7.00	40.00	0.00
Fusarium semitectum Berkeley & Ravenel	Sem	7.00	50.00	2.00
Fusarium solani (Martius) Appel & Wollenweber emend. Snyder & Hansen	Sol	2.00	40.00	14.00
Fusarium sporotrichioides Sherbakoff	Spo	94.00	400.00	8.00
Fusarium tricinctum (Corda) Saccardo	Tri	0.00	50.00	0.00
Fusarium verticillioides (Saccardo) Nirenberg	Ver	3.00	0.00	1.00
Fusarium spp.	spp	54.75	250.00	42.00

Table S5. Dominance structure as number of colony forming units of *Fusarium* species detected on the body surface of collected arthropods per individual, averaged per sampling point. Abbreviations of the *Fusarium* species are explained in Table S4.

Kettle hole ID Sampling point	Ave	Cer	Cul	Dim	Equ	Gra	Mer	Oxy	Poa	Sam	Sci	Sem	Sol	Spo	Ver	spp.
163C	1.50	0.50	26.63	0.00	0.00	0.00	0.00	0.00	0.00	1.25	0.00	1.00	0.00	4.50	0.50	1.00
166A	1.00	0.00	0.33	0.17	0.33	0.33	0.00	0.00	0.00	1.67	0.17	0.33	0.00	1.00	0.17	2.33
166B	0.00	0.00	0.00	0.00	0.00	0.00	0.00	0.00	0.00	0.00	0.00	0.00	0.00	0.00	0.00	0.00
166C	0.00	0.00	0.00	0.25	0.00	0.00	0.00	0.00	0.00	0.00	0.00	0.00	0.00	0.00	0.00	0.25
172A	0.00	0.00	0.00	0.67	0.00	0.00	0.00	0.00	0.00	0.33	0.00	0.00	0.33	0.33	0.00	1.33
807A	0.00	0.00	1.14	1.29	0.00	0.00	0.00	0.00	0.00	0.00	0.00	0.00	0.14	0.29	0.00	0.00
807B	0.00	0.00	9.00	0.40	0.20	0.00	0.00	0.00	0.00	0.00	0.00	0.00	0.00	0.60	0.00	0.00
807C	0.00	0.00	1.00	0.17	0.33	0.17	0.00	0.33	0.00	0.00	0.00	0.00	0.00	0.33	0.00	0.67
808A	0.00	0.00	0.20	0.30	0.00	0.10	0.00	0.00	0.00	0.95	0.00	0.20	0.00	1.20	0.10	0.00
808B	0.00	0.00	0.50	0.25	0.00	0.00	0.13	0.13	0.00	0.13	0.00	0.00	0.00	0.13	0.00	0.13
808C	0.17	0.00	0.00	0.33	0.00	0.00	0.00	0.25	0.00	0.25	0.00	0.00	0.00	0.00	0.00	0.00
1172A	2.33	0.00	0.33	0.33	0.00	0.00	0.00	0.33	0.00	1.67	0.00	0.00	0.00	15.67	0.00	2.00
1172B	0.67	0.33	7.00	0.00	1.00	0.00	0.00	0.00	0.00	1.83	1.33	0.00	0.00	1.33	0.00	2.33
1172C	0.00	0.00	0.20	0.20	1.00	0.00	0.20	0.20	0.20	1.55	0.40	0.20	0.00	1.20	0.00	1.33
1192C	0.00	0.00	3.00	1.00	0.00	0.00	3.00	2.00	0.00	3.00	0.00	0.00	0.00	1.00	0.00	0.00

Table S6. Dominance structure as number of colony forming units of *Fusarium* species detected in 1.0 mg dry mass of litter samples. Abbreviations of the *Fusarium* species are explained in Table S4.

Kettle hole ID Sampling point	Ave	Cul	Dim	Gra	Mer	Poa	Pro	Sam	Sci	Sem	Sol	Spo	Tri	spp.
163A	0	0	0	0	0	0	0	0	0	0	0	20	20	0
163B	30	0	70	0	0	0	0	0	0	0	10	0	0	0
163C	0	70	0	0	0	0	0	10	10	10	0	0	0	0
166A	0	0	270	0	0	0	0	20	10	0	0	0	0	0
166B	0	0	10	0	50	0	0	0	0	0	0	0	0	0
166C	0	0	0	0	0	10	0	0	0	0	0	10	0	0
172A	10	0	0	0	0	0	0	30	0	0	10	60	0	0

172B	0	40	0	0	0	0	0	0	0	0	0	60	0	100
172C	0	20	0	0	10	0	0	0	0	0	0	20	0	0
807A	0	0	0	0	0	0	0	0	0	0	0	30	0	0
807B	0	40	0	0	0	0	0	0	0	0	0	40	0	0
807C	0	0	0	0	0	0	0	0	0	0	0	10	0	0
808A	0	0	0	0	0	0	20	0	0	0	0	0	0	0
808B	0	10	0	0	0	0	30	10	10	0	0	20	0	0
808C	0	560	0	0	0	0	0	100	0	0	10	0	0	0
1172A	0	0	0	0	0	0	0	20	0	0	0	10	0	0
1172B	0	0	0	0	0	0	0	0	0	0	0	10	0	0
1172C	0	0	0	0	0	0	0	0	0	0	0	0	20	0
1192A	30	0	0	0	0	0	0	0	10	0	0	70	10	20
1192B	0	0	0	0	0	0	0	0	0	0	0	0	0	0
1192C	20	0	0	0	20	0	0	50	0	40	10	40	0	130

Table S7. Dominance structure as number of colony forming units of *Fusarium* species detected in 1.0 mg dry mass of soil samples. Abbreviations of the *Fusarium* species are explained in Table S4.

Kettle hole ID Sampling point	Cer	Cul	Dim	Gra	Mer	Oxy	Poa	Pro	Sam	Sem	Sol	Spo	Ver	spp.
163A	1	0	0	0	3	0	0	0	1	0	2	0	0	0
163B	0	3	0	0	2	0	1	0	1	0	3	3	1	2
163C	0	0	0	0	0	0	0	0	0	0	0	0	0	4
166A	0	0	0	0	0	0	0	0	1	0	0	0	0	1
166B	3	3	0	0	0	0	0	0	4	0	0	0	0	3
166C	0	1	1	0	0	0	0	0	1	0	1	1	0	2
172A	0	4	2	0	0	0	0	0	2	0	0	0	0	0
172B	0	4	0	0	0	1	0	0	1	0	1	0	0	0
172C	0	3	0	0	0	0	0	0	0	0	0	2	0	0
807A	0	3	0	0	0	0	0	1	0	0	0	0	0	1
807B	0	1	0	0	0	0	0	0	0	0	0	0	0	4
807C	3	5	0	0	2	0	0	0	1	0	0	0	0	4

808A	0	1	0	0	0	0	0	0	2	0	3	2	0	0
808B	1	1	0	0	0	0	0	0	0	0	1	0	0	0
808C	1	1	0	0	1	0	0	0	1	0	0	0	0	3
1172A	0	4	0	0	0	0	0	0	0	1	0	0	0	5
1172B	0	0	0	0	0	0	0	0	2	0	0	0	0	9
1172C	0	2	0	0	0	0	0	0	1	0	1	0	0	0
1192A	0	1	0	0	0	0	0	0	0	0	0	0	0	3
1192B	1	0	0	1	0	1	0	0	0	1	1	0	0	1
1192C	0	0	0	0	0	0	0	0	0	0	1	0	0	0

1. Braun-Blanquet, J. Pflanzensoziologie: Grundzüge der Vegetationskunde, 2nd ed. Springer- Verlag: Wien, Austria, 1951.
